# The temporal network of mobile phone users in Changchun Municipality, Northeast China

**DOI:** 10.1038/sdata.2018.228

**Published:** 2018-10-30

**Authors:** Zhanwei Du, Yongjian Yang, Chao Gao, Liping Huang, Qiuyang Huang, Yuan Bai

**Affiliations:** 1 College of Computer Science and Technology, Jilin University, 130012, Changchun, China; 2 Department of Integrative Biology, University of Texas at Austin, Austin, 78705, USA; 3 College of Computer and Information Science & College of Software, Southwest University, Chongqing 400715, China

**Keywords:** Geography, Civil engineering

## Abstract

Mobile data are a feasible way for us to understand and reveal the feature of human mobility. However, it is extremely hard to have a fine-grained picture of large-scale mobility data, in particular at an urban scale. Here, we present a large-scale dataset of 2-million mobile phone users with time-varying locations, denoted as the temporal network of individuals, conducted by an open-data program in Changchun Municipality. To reveal human mobility across locations, we further construct the aggregated mobility network for each day by taking cellular base stations as nodes coupled by edges weighted by the total number of users’ movements between pairs of nodes. The resulting temporal network of mobile phone users and the dynamic, weighted and directed mobility network are released in simple formats for easy access to motivating research using this new and extensive data of human mobility.

## Background & Summary

Building on advances in fields such as the wireless communication and high-performance computation, scientists are enabled to collect position information of individuals accessing human movements. Based on such information, the feature of human mobility is revealed and characterized, which has widespread applications (e.g., population mobility^1–3^, activity patterns^4–6^, urban evolution^7,8^, mobile virus propagation^9^, and temporal network epidemiology^10^) and inspire other similar research fields, such as mobile phone viruses^11^.

To study human movements, the conceptual representation of the individual network is commonly studied to characterize interactions among people. A bulk of empirical and theoretical/computational network studies have been made, most if not all of which are static networks oriented^12–14^, neglecting the fact that human movements are dynamic. Temporal networks provide a new modeling and data analysis framework by taking into account the time ordering of interactions^15–17^. A body of related datasets, such as wifidog^18^ and mobility evolution^19^, are released under this framework for real-time monitoring of people’s behaviors. However, although studies^1,20–22^ focus on the fine-grained data of large-scale population for the temporal network, the high-resolution datasets of individual movements along time are still not open with easy access due to potential reasons (e.g., the economic cost and data privacy).

In this paper, we integrate the fine-grained data via real-time monitoring of mobile phones from an open-data program in Changchun Municipality. The temporal network of people is compiled via sequential hourly snapshots of mobile phone users’ positions. The dataset contains the movements of over 2,066,000 anonymized mobile phone users in each day between 7,251 cellular base stations in Changchun municipality area during a one-week period since 3 July 2017. This municipality area comprising seven districts had the population of over 4,378,000 in an area of 7,557 square kilometers in 2016, which is the core city of Northeast Asia (http://www.china.org.cn/english/features/43592.htm).

Note that there are areas with high populations, as this more than one cellular base stations may locate in the same place to provide services. We cluster these nearby cellular base stations into 3,406 clusters (henceforth locations). Each daily movement trace is represented by 24-dimension location vector for 24 sequential hours in a day, denoting locations individual stayed at last in each hour. From these data, the group of users in the same location and time can be considered as having interactions with each other by associating users to their spatial locations as their current staying locations in the same period. We additionally construct a dynamic mobility network for each day in favor of time-aggregated representations. The locations indicate nodes, while users’ movements between the pair of nodes along the day as edges.

To facilitate handling of the open data, we save the above information on the temporal network of individuals, as well as the mobility network of locations in the standard format files, separated by commas. There are 15 files released in 3 folders. For each day of the studied week, there are two corresponding files. One file denotes the temporal network with rows of hourly staying location in a day for each mobile phone user. Moreover, another one represents the mobility network, contains three columns ordered by origin location, destination location, and their weight. In each day, there are more than 30 million movements of over 2 million individuals in the daily temporal network and more than 900,000 edges of over 3,000 nodes in the mobility network. For future potential spatial analysis, we also provide an additional file denoting the distance between any pair of locations, containing three columns ordered by origin location, destination location and their distance in the kilometer. Although the dataset covers a cohort of millions of users, it is only one week period in summer. This discrepancy can induce a bias in the study of mobility flows, in contrast with long-term observations. Researchers should be aware of this potential limitation using this dataset.

## Methods

### Original data sources

Our data consist of one-week location records of anonymized mobile phone users in Changchun Municipality, since 3 July 2017. Each mobile phone is uniquely identified. A cell phone can be located to the closest cellular base station via tracking its most recent sending and receiving signals. As thus, each location record is associated with a time stamp. If a phone is failed to be located and even corrected, it is recorded as the missing status, denoted ‘0’. For each user, we derive the corresponding location records into movement traces to represent the hourly time series of locations. Each movement trace contains the time series of locations, where this individual stayed the last in each period. Sometimes, a user may visit lots of locations in an hour. For example, a user is tracked with 3 locations (e.g., *a*, *b*, and *c*) in the day #. Location *a* emerges in 8:30, *b* in 8:45, and *c* in 8:50. We used the last location *c* to label this user’s location for the hour between 08:00 to 08:59. The location where one stays for the last time is regarded as the user’s location. Finally, each user is mapped to a location in an hour. Each location is identified by an anonymized identification code. The telecommunications operator kindly accepted to grant us the rights of sharing these anonymized movement traces and licensing this derived dataset in the framework of the temporal network as Open Data under Attribution 4.0 International (CC BY 4.0) license. The temporal networks of mobile phone users are released via location snapshots for each user for each hour of a week. There are 7,251 cellular base stations. We release the raw files of hourly temporal networks containing the information available in the figshare websites during the studied week.

### Location correction

The original movement data take each base station as a unique unit. Note that several cellular base stations may locate in the same place to provide services together. We cluster cellular base stations together as one location if they are quite near to each other. A cluster of stations, as a new location, is identified as a group of stations in which the distance between any pair of stations is less than 100 meters. For the clustering approach, we construct a network of cellular base stations as nodes. The edge between a pair of nodes denotes their distance is no more than 100 meters. Nodes connected by edges are identified as the same location and labeled by the same location ID.

Users can only be located when they send or receive signals, connecting to the closest cellular base station. As thus the individual movement trace is sparse with only several available positions in a day. If a user is failed to track in an hour, we use ‘0’ to label this user’s position in this hour.

### Defining the mobility network

Users usually appear in the same location and time. We can associate them together as a group, labeled as their shared spatial location. To facilitate the common analysis at location level, we additionally construct a dynamic mobility network via daily snapshots. A movement from location *i* to location *j* denotes in a user’s daily movement trace, the location *j* emerges after the previous tracked location *i*. The nodes of the mobility network denote the locations, and the edges represent the mobility between the pair of nodes, weighted by the aggregation of the total number of movements between an origin node and a destination node along the day.

### Code availability

Matlab codes for data analysis of location correction and mobility network construction can be obtained freely by contacting the authors, with no restrictions to access.

## Data Records

This dataset is released by comma-separated values (CSV) files in 3 folders at over 2,066,000 anonymized mobile phone users for each day (Data Citation 1). There are two files for each day. One file records the temporal network with rows of locations for each node to denote the movements in a day. Another infers the mobility network with rows of origin location, destination location, and their weight. The weight is the number of movements between the origin location, destination location in the studied day. In each day, there are over 3,000 nodes with more than 900,000 in the temporal network and over 3,000 nodes with more than 900,000 edges in the mobility network. We provide an additional file recording the distances for all pairs of locations with three columns as the origin location, destination location and their distance in the kilometer.

Finally, three folders are used to group these files (Data Citation 1). The first folder (**Day-#-temporal**) includes seven files (**Day-#-temporal.txt**) of the temporal network for each day of the week to track each individual. The second folder (**Day-#-mobility**) includes 7 files (**Day-#-mobility.txt**) of mobility network with locations as nodes. The third folder (**Distance-locations**) contains one file to denote the distances between locations.

**Day-#-temporal.txt** In the temporal network, each row represents a daily movement pattern of 24 hours in a day. The format for this file is the following: *n*, *h*01, *h*02, …, *dn*. The identification for each user is removed, which retains the daily and weekly patterns of human mobility. The anonymization of users’ identification can cut users’ sequential position tracking across days and avoid potential re-identification of users by illegal attacks, which could use individuals’ long-period traces to identify unique users^23^.

- *n*: number of users following this daily movement pattern;
- h-##: location ID of a user in the hour of starting from ##:00 to ##:59 in the day #. When there is no available location information in this hour, we denote this status as ‘0’;

**Day-#-mobility.txt** In the mobility network, the daily mobility flow *F* is studied, with the entry of *F*_*ij*_ as the mobility flow as the edge weight between the *i*-th origin node and the *j*-th destination node. Specifically, each row represents an edge, weighted by the aggregation of the number of hourly movements between an origin node and a destination node along the day. A movement in the hour ## from location *i* to location *j* denotes that location *j* emerged in the hour ## after location *i*. For example, if there are users visiting between location *i* and location *j* for 100 times over hours in this day, *F*_*ij*_ is counted as 100.

- origin: numerical ID for each origin node;
- destination: numerical ID for each destination node;
- weight: number of movements between origin location, destination location in the day #;

**Distance-locations.txt** The distance set *D* between all pairs of locations. The format for this file is organized as three columns. In each row, there are three values, denoting the origin location with numerical id as *i*, the detonation location with numerical id as *j* and their distance *d*_*ij*_, receptively.

- origin: numerical ID for each origin node;
- destination: numerical ID for each destination node;
- distance: great-circle distance estimated by the haversine formula in kilometer between each pair of the origin location and destination location in the day #;

## Technical Validation

**Figure 1:**
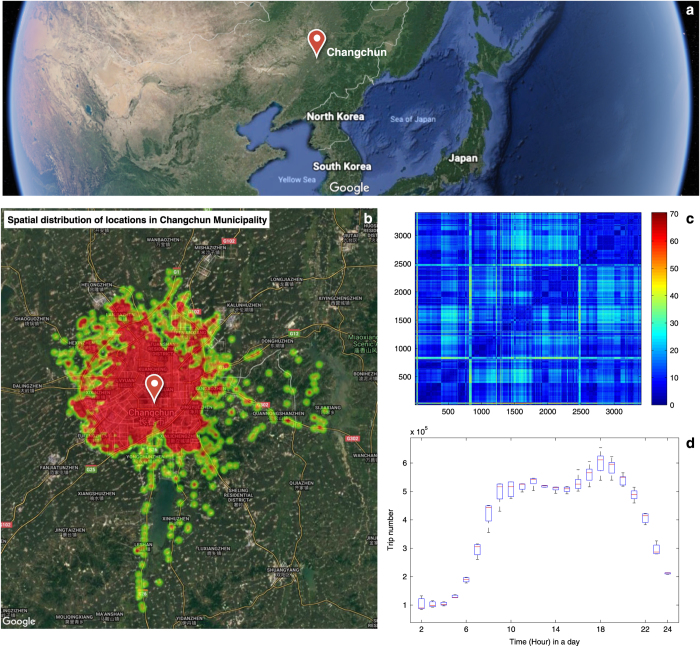
Spatial distribution of locations. (**a**) The geographical position of Changchun Municipality in the earth view, as the core city of Northeast Asia. (**b**) The geographical heat map of 3,406 locations in Changchun, the horizontal axis denotes the latitudes of locations. The vertical axis denotes the longitude of locations. The spatial map was created using Google map. (**c**) The heat map of distances (kilometers) between 3,406 locations. The rows (or columns) of this heat map represent locations. Each cell is colorized based on the level of distance between locations. The redder the color is, the longer the distance is. (**d**) The movements between different locations in the studied week. A movement in the hour ## from location *i* to location *j* denotes that location *j* emerged in the hour ## after location *i*. The x-axis denotes 23 h in a day starting from 2 am. The y-axis denotes the total number of movements which have a different location in contrast with the last locations. For each hour, we estimate the first and third quartiles of the number of these movements over seven days of the week. There are two peaks in a day, i.e., morning peak starting at 9 am and evening peak starting at 5 pm.

The reliability of users’ movements in location and time largely depends on the reliability of the source data. We verify the consistency of the location correction procedure by visualizing locations on the distribution of locations’ distances. We visualize them on a geographic map of 3,406 locations, as shown in Fig. 1, as well as their distances between locations. More positions are in the center of the city, whose distances below 40 kilometers.

### Temporal network

We verify the consistency of the temporal network with people’s daily life with the hourly temporal flows over seven days of the week, as shown in Fig. 1. A trip denotes an individual movement, whose origin node is different from its destination node. For each hour, we count the number of trips with changing locations as the hourly trip flow. The hourly trip flows of all working days show two traffic peaks (morning and evening). The morning period is starting at 9:00, and the evening is beginning at 17:00. Both are approximately 4 h long, similar to the mobility flows in another Chinese city of Shanghai with the morning period starting at 9 am, and the evening period starting 4 pm^4^.

**Figure 2:**
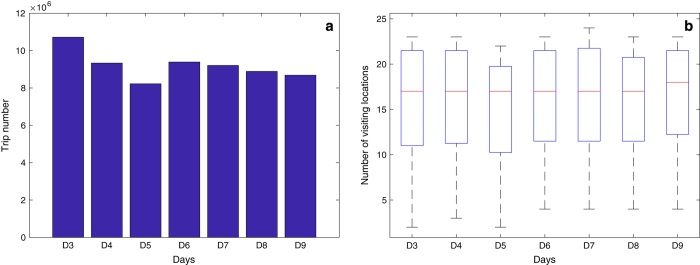
Daily trip numbers and individual average visiting location number. X axis denotes the 7 days of week. D# infers the #-th day. (**a**) Y axis represents the total trip number. (**b**) Y axis denotes the average visiting location number without information per user for each day. For example of D3, it, as for Monday, is featured by the highest trip number, as well as the average visiting locations per user.

To investigate users’ movement patterns regarding time usage and location visited, we evaluate for the visiting location number per user and the trip number for each day of the studied week (Fig. 2). Monday is a traditional weekday with the highest traffic flows, similar as in other Chinese traffic systems (such as Beijing, Shanghai, Guangzhou, and Shenzhen (https://zhuanlan.zhihu.com/p/25432609). As for Wednesday, it is accidentally impacted by a large-scale scheduled power outages in 10 regions of this city, especially during 8:00 to 10:00 (http://www.dianping.com/toutiao/935312), decreasing mobility flows to some extent. We find that the number of trips in this dataset is slightly higher on Monday compared to other days, especially Wednesday.

### Mobility network

**Figure 3:**
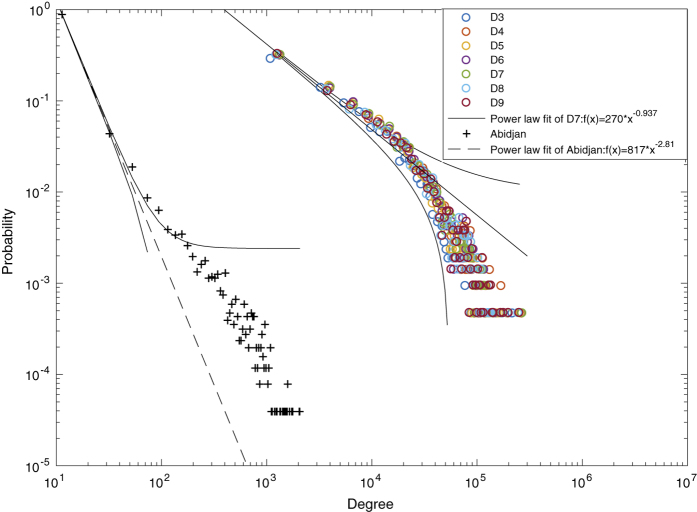
Degree distribution. For the mobility network of locations as nodes, we estimate the degrees of nodes as the total number of hourly movements between different locations along the day. The probability of degree distribution is shown over seven days of the studied week. D# infers the #-th day. The probability distributions are similar with each other, following the power-law distributions *f*(*x*)=*a* ∗ *x*^*b*^, where *x* is the degree and *f*(*x*) is the corresponding probability. For example of Sunday (D9), the fitted power law distribution is validated by the chi-square goodness-of-fit test, with *r*^2^ as 0.99. Specifically, *a* = 270 within 95% confidence bounds between 210.1 and 331.9, and *b* = −0.937 within 95% confidence bounds between −0.967 and −0.9079. In contrast, we study the degree distribution of the static mobility network with 381 cell phone antennas, aggregating 607,167 mobile phone users’ two-week movements in Abidjan (the biggest city of Ivory Coast)^12^. We can see this distribution follows the power law distribution with *r*^2^ as 0.99. Here, *a* = 817 within 95% confidence bounds between 728.4 and 906.5, and *b* = −2.811 within 95% confidence bounds between −2.856 and −2.766. The smaller *b* denotes fewer locations contributing to most movements flows than that of Changchun.

In the mobility network, nodes are defined as locations, as well as edges weighted by the mobility flows between nodes. We additionally analyze the mobility network via its topology features. The degrees of nodes denote the total number of hourly movements between different locations along the day. For example, the degrees of 3,406 nodes are shown in Fig. 3, whose shape of the degree probability distribution also correctly follows the power law distribution. In contrast, the degree distribution of the static mobility network in Abidjan^12^ also follows the power law distribution as *f*(*x*)=817 ∗ *x*^−2.81^, with fewer locations contributing to most flows than that of Changchun.

The relationship between the mobility flow *F* and traveling distance *D* for each pair of locations are studied. Specifically, we estimate the Pearson’s correlation coefficients between *log*(1/*D*^2^) and *log*(*F*) with *P*<0.05. For example of the day of *D*3, the Spearman’s correlation coefficient is 0.43, denoting the strong correlation, similar in other six days. Additionally, the gravity mobility model^24^ assume that the relative attraction as $A_{s->j}∼m_{s}m_{j}/d_{sj}^{2}$, with origin population *m*_*s*_, destination population *m*_*j*_ and distance *d*_*sj*_ between two stations *s* and *j*. In the mobility network, we assume *m*_*s*_ as the total number of users coming to location *s* as $\sum_{i}F_{is}$, and *m*_*j*_ as $\sum_{i}F_{ij}$. We evaluate the correlation of *A*_*s*->*j*_ and $F_{sj}/\sum_{k\neq j}^{}F_{kj}$ over pairs of different locations. For example of the day of *D*3, the Spearman’s correlation coefficient is 0.51 with *P* < 0.05, similar in other six days, denoting obvious connections between mobility flows and distances.

## Additional information

**How to cite this article**: Pal, A. *et al*. High content organelle trafficking enables disease state profiling as powerful tool for disease modelling. *Sci. Data*. 5:180228 doi: 10.1038/sdata.2018.228 (2018).

**Publisher’s note**: Springer Nature remains neutral with regard to jurisdictional claims in published maps and institutional affiliations.

## Supplementary Material
